# Arnica Tincture Cures Cutaneous Leishmaniasis in Golden Hamsters

**DOI:** 10.3390/molecules23010150

**Published:** 2018-01-12

**Authors:** Sara M. Robledo, Ivan D. Vélez, Thomas J. Schmidt

**Affiliations:** 1 PECET-School of Medicine, University of Antioquia, Calle 70 # 52-21, 0500100 Medellin, Colombia; sara_robledo@yahoo.com or sara.robledo@udea.edu.co (S.M.R.); id_velez@yahoo.com or ivan.velez@udea.edu.co (I.D.V.); 2 Institute of Pharmaceutical Biology and Phytochemistry (IPBP), University of Münster, PharmaCampus-Corrensstrasse 48, D-48149 Münster, Germany

**Keywords:** *Arnica montana* L., cutaneous Leishmaniasis, neglected tropical disease

## Abstract

In search for potential therapeutic alternatives to existing treatments for cutaneous Leishmaniasis, we have investigated the effect of Arnica tincture Ph. Eur. (a 70% hydroethanolic tincture prepared from flowerheads of *Arnica montana* L.) on the lesions caused by infection with *Leishmania braziliensis* in a model with golden hamsters. The animals were treated topically with a daily single dose of the preparation for 28 days. Subsequently, the healing process was monitored by recording the lesion size in intervals of 15 days up to day 90. As a result, Arnica tincture fully cured three out of five hamsters while one animal showed an improvement and another one suffered from a relapse. This result was slightly better than that obtained with the positive control, meglumine antimonate, which cured two of five hamsters while the other three showed a relapse after 90 days. This result encourages us to further investigate the potential of Arnica tincture in the treatment of cutaneous Leishmaniasis.

## 1. Introduction

Infections caused by unicellular parasites of the genus *Leishmania* represent a world-wide problem. Leishmaniasis, depending on the species causing it can occur either as a visceral disease affecting the inner organs, or as a cutaneous or mucocutaneous form affecting the skin and/or moucous membranes. While visceral Leishmaniasis (VL), mainly cause by *Leishmania infantum* (Americas) and *L. donovani* (old world) often is a lethal disease if untreated, cutaneous Leishmaniasis (CL, caused by about 20 different species including *L. braziliensis*) may cause very severe skin lesions that are difficult to treat, heal only very slowly and may lead to dramatic disfiguration of the patients. In case of the very severe mucocutaneous form, most often caused by *L. braziliensis*, patients may eventually suffer from a complete destruction of the face including mouth and nasopharynx. Only few efficacious, safe and affordable therapies exist for Leishmaniasis so that it represents a very severe problem in many tropical countries, but increasingly also outside the tropics. Due to lack of investment into the development of new drugs, Leishmaniasis has been classified a neglected tropical disease (NTD) by the world health organization (WHO).

In the course of our search for potential new treatment options for NTDs, we recently reported on a screening of various legally approved herbal medicinal preparations and their in vitro activity against protozoan parasites [1]. One of the preparations tested in this work was a commercial Arnica tincture which showed high activity against *Trypanosoma brucei* but also considerable growth inhibitory activity on *Leishmania donovani*, one of the pathogens causing visceral Leishmaniasis. In previous studies, considerable in vitro activity against protozoan parasites had been reported for single consitutents of Arnica, namely, sesquiterpene lactones (STLs) of the helenalin type [2,3,4]. Arnica tincture and other preparations from the same plant material are commonly used as safe and efficacious topical treatment for inflammatory conditions of the skin, joints or muscles [5] which has been mainly attributed to its STLs and their inhibitory activity on inflammatory transcription factor NF-κB [6]. On the other hand, internal treatment is not recommended due to a risk of toxic side effects [7]. We therefore decided to investigate whether Arnica tincture might serve as a topical treatment for CL. The current study is a first report on the results of an in vivo study on the effect of Arnica tincture on *L. braziliensis* infection in golden hamsters.

## 2. Results and Discussion

### 2.1. Chemical Composition of the Tested Arnica Tincture

**Figure 1:**
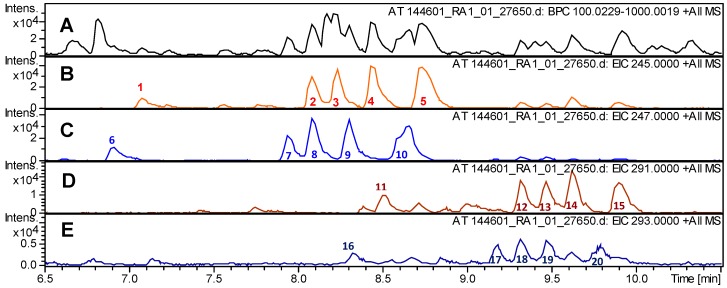
Ultra high performance liquid chromatography/positive mode electrospray ionization quadrupole time of flight tandem mass spectrometry (UHPLC/+ ESI QqTOF MSMS) analysis of the investigated Arnica tincture; retention time range relevant for sesquiterpene lactones (STLs). (**A**) Base peak ion chromatogram (*m*/*z* 100–1000); (**B**) Extracted ion chromatogram *m*/*z* 245 [C_15_H_17_O_3_]^+^, representing the main common fragment of helenalin esters; (**C**) Extracted ion chromatogram *m*/*z* 247 [C_15_H_19_O_3_]^+^, representing the main common fragment of 11α,13-dihydrohelenalin esters; (**D**) Extracted ion chromatogram *m*/*z* 291 [C_17_H_23_O_4_]^+^, representing the main common fragment of 2-ethoxy-2,3-dihydrohelenalin esters; (**E**) Extracted ion chromatogram *m*/*z* 293 [C_17_H_25_O_4_]^+^, representing the main common fragment of 2-ethoxy-2,3,11,13-tetrahydrohelenalin esters. The compounds detected in (**D**,**E**) are artefacts known to be formed in Arnica tincture by Michael addition of ethanol to the cyclopentenone unit of the native STLs [8]; for peak identification see Figure 2.

**Figure 2:**
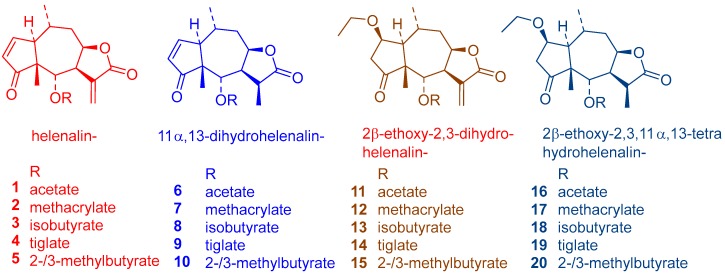
Sesquiterpene lactones identified in the Arnica tincture under study.

The investigated Arnica tincture obtained from a commercial source was analyzed by ultra high performance liquid chromatography/positive mode electrospray ionization quadrupole time of flight tandem mass spectrometry (UHPLC/+ ESI QqTOF MSMS). The main constituents, sesquiterpene lactones (STLs) of the helenalin- and 11α,13-dihydrohelenalin series, were identified by their exact masses and in accordance with reported literature data [8]. The identified STLs are labelled in the chromatogram depicted in Figure 1 and their chemical structures are shown in Figure 2. The total content of sesquiterpene lactones presumably responsible for the overall activity was approximately determined to be 0.047% and was thus in agreement with the content by the prescribed by the European Pharmacpoeia [9].

### 2.2. In Vivo Effect of Arnica Tincture on L. braziliensis Infection in Golden Hamsters

**Figure 3:**
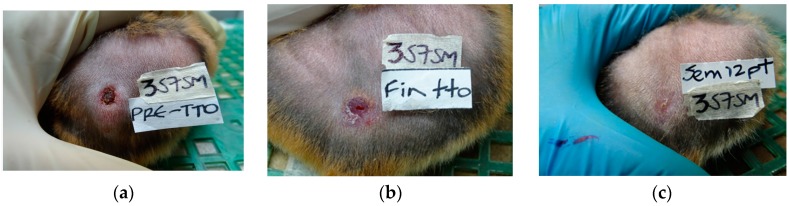
Treatment progress of cutaneous leishmaniasis caused by *L. braziliensis* in one animal (4AE-357-SM-♀ in Table 1). (**a**) Before treatment (TD0); (**b**) On day 28 at the end of treatment (TD28); (**c**) on day 90 post treatment (PTD90).

Golden hamsters were infected with *L. (V) braziliensis*. After full development of skin lesions, they were treated for 28 days with a single daily dose (100 µL/lesion, corresponding to a dose of approximately 50 ng STLs/treatment) of Arnica tincture or with a single dose (200 μg in 100 μL) of meglumine antimoniate (Glucantime^®^) twice a week during four weeks. A third group of hamsters was left without treatment. Lesion size was measured according to the width and length of the ulcer using a digital caliper every two weeks during the time of treatment and monitored further until day 90 after the end of the treatment. Body weight was also recorded during the study. The results for Arnica tincture and for the positive (meglumine antimoniate) and negative (untreated) controls are reported in Table 1. The healing process of one of the animals treated with Arnica tincture is shown in Figure 3.

In case of the standard drug, three of five animals did not show any lesions on the last day of treatment (TD28) and all five were apparently cured 15 days after the end of the treatment. However, three of the five animals in this group showed a relapse so that only two were considered as cured on post-treatment day (PTD) 90. Arnica tincture led to a decrease of lesion size on TD28 in three animals but the symptoms then re-appeared on post-treatment day PTD15 reaching a maximum on PTD45. Interestingly, the lesions then gradually decreased in size so that on PTD90, three of five hamsters showed a complete cure while one animal showed a significant improvement and only the fifth one still suffered from a relapse. Thus, Arnica tincture led to a 60% rate of complete cure while this was only 40% in case of the antimonial standard drug. No cure was observed in hamsters that remained without treatment, i.e., all of them suffered from severe lesions of active CL. The different course of development observed with Arnica tincture and meglumine antimoniate (i.e., slower progress of healing and intermediate re-increase of lesion size followed by complete healing in case of the former, faster and continuous decrease in case of the latter) may possibly be explained by the different ways of administration. The antimonial drug is required to be administered by injection into the dermis within in the lesion whereas the tincture was applied topically, i.e., to the surface of the ulcer so that the onset of a therapeutic effect may have been slowed down. It should be noted that intralesional injection is a much more painful treatment so that a drug that could be applied topically, as would be feasible with Arnica tincture, would also represent a benefit in this respect. Photographic documentation of all animals’ lesion development is provided as supplementary material (Figures S1–S3).

**Figure 4:**
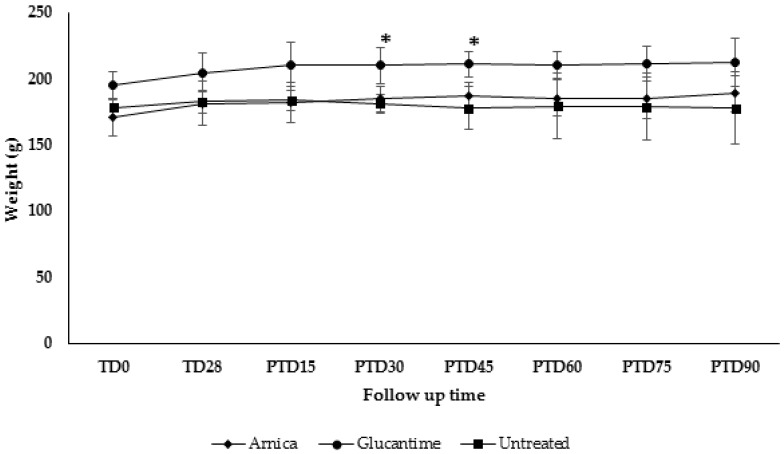
Effect of treatment in the body weight. Golden hamster (*n* = 5 each group) were treated with Arnica tincture or meglumine antimoniate (Glucantime^®^) during four weeks, administered topically or intralesionally, respectively. Untreated hamsters were also supervised during the study. Data represents the mean value ± SD of the weight in grams of hamsters in each experimental group. * *p* < 0.05.

Arnica tincture treatment did not produce any detrimental effect on the body weight in most hamsters, but rather a slight weight gain was observed in most of them. Only one hamster (3AE-356-OD-♂) did show a weight loss of 1.2% at PTD60. Similar results were observed in hamsters treated with Glucantime^®^ but in this case, the animals suffered from a slight weight loss of 0.98%, 3.93% and 7.84% at days 60, 75 and 90 post treatment (Figure 4). Statistically significant differences were only observed in hamsters treated with meglumine antimoniate from day 60 to 75 post treatment.

## 3. Materials and Methods

### 3.1. Tested Materials

Arnica tincture is a legally approved herbal drug in Germany. The preparation used in this study was Arnikatinktur Hetterich (PZN 3060698), manufactured by Teofarma srl, Valle Salimbene, Italy. Glucantime^®^ was obtained from Sanofi-Aventis (São Paulo SP, Brazil).

### 3.2. Chemical Analysis

#### UHPLC/+ ESI QqTOF MSMS

High-resolution mass determinations were performed on a Dionex (Idstein, Germany) Ultimate 3000 RS Liquid Chromatography System on a Dionex Acclaim RSLC 120, C18 column (2.1 mm × 100 mm, 2.2 µm) with a binary gradient (A: water with 0.1% formic acid; B: acetonitrile with 0.1% formic acid) at 0.8 mL/min: 0 to 9.5 min: linear from 5% B to 100% B; 9.5 to 12.5 min: isocratic 100% B; 12.5 to 12.6 min: linear from 100% B to 5% B; 12.6 to 15 min: isocratic 5% B. The injection volume was 2 µL. Eluted compounds were detected using a Dionex Ultimate DAD-3000 RS over a wavelength range of 200–400 nm and a Bruker Daltonics (Bremen, Germany) micrOTOF-QII time-of-flight mass spectrometer equipped with an Apollo electrospray ionization source in positive mode at 5 Hz over a mass range of *m*/*z* 50–1000 using the following instrument settings: nebulizer gas nitrogen, 5 bar; dry gas nitrogen, 9 L/min, 220 °C; capillary voltage 4500 V; end plate offset −500 V; transfer time 70 µs; collision gas nitrogen; collision energy and collision RF settings were combined to each single spectrum of 1000 summations as follows: 250 summations with 20% base collision energy and 130 Vpp + 250 summations with 100% base collision energy and 500 Vpp + 250 summations with 20% base collision energy and 130 Vpp + 250 summations with 100% base collision energy and 500 Vpp. Base collision energy was 50 eV for precursor ions with a *m*/*z* less than 500 and then linearly interpolated against *m*/*z* up to a maximum of 70 eV for precursor ions with a *m*/*z* of up to 1000. Internal dataset calibration (HPC mode) was performed for each analysis using the mass spectrum of a 10 mM solution of sodium formiate in 50% isopropanol that was infused during LC re-equilibration using a divert valve equipped with a 20 µL sample loop. Undiluted Arnica tincture (injection volume 2 µL) was used for the analysis.

High-resolution mass determinations were performed on a Dionex (Idstein, Germany) Ultimate 3000 RS Liquid Chromatography System on a Dionex Acclaim RSLC 120, C18 column (2.1 mm × 100 mm, 2.2 µm) with a binary gradient (A: water with 0.1% formic acid; B: acetonitrile with 0.1% formic acid) at 0.8 mL/min: 0 to 9.5 min: linear from 5% B to 100% B; 9.5 to 12.5 min: isocratic 100% B; 12.5 to 12.6 min: linear from 100% B to 5% B; 12.6 to 15 min: isocratic 5% B. The injection volume was 2 µL. Eluted compounds were detected using a Dionex Ultimate DAD-3000 RS over a wavelength range of 200–400 nm and a Bruker Daltonics (Bremen, Germany) micrOTOF-QII time-of-flight mass spectrometer equipped with an Apollo electrospray ionization source in positive mode at 5 Hz over a mass range of *m*/*z* 50–1000 using the following instrument settings: nebulizer gas nitrogen, 5 bar; dry gas nitrogen, 9 L/min, 220 °C; capillary voltage 4500 V; end plate offset −500 V; transfer time 70 µs; collision gas nitrogen; collision energy and collision RF settings were combined to each single spectrum of 1000 summations as follows: 250 summations with 20% base collision energy and 130 Vpp + 250 summations with 100% base collision energy and 500 Vpp + 250 summations with 20% base collision energy and 130 Vpp + 250 summations with 100% base collision energy and 500 Vpp. Base collision energy was 50 eV for precursor ions with a *m*/*z* less than 500 and then linearly interpolated against *m*/*z* up to a maximum of 70 eV for precursor ions with a *m*/*z* of up to 1000. Internal dataset calibration (HPC mode) was performed for each analysis using the mass spectrum of a 10 mM solution of sodium formiate in 50% isopropanol that was infused during LC re-equilibration using a divert valve equipped with a 20 µL sample loop. Undiluted Arnica tincture (injection volume 2 µL) was used for the analysis.

The approximate content of STLs in the investigated Arnica tincture was also determined with this UHPLC/DAD/MS setup using a modified method based on the HPLC method of the European Pharmacopoeia (Ph. Eur. 9) [9]. UHPLC conditions were as described above. The tincture samples were treated as prescribed, α-Santonin was used as internal reference compound and the quantification based on the UV-absorbance detected at λ = 225 nm as prescribed by the Ph. Eur. monograph. All peaks assigned to STLs were integrated and the total contents calculated according to the Ph. Eur. monograph. Two independent analyses were performed yielding a mean of 0.047 ± 0.001% of total STLs.

### 3.3. Animal Experiments

Male and female golden hamsters (*Mesocricetus auratus*), six or seven week-old, were infected with 5 × 10^7^ stationary growth phase promastigotes of *L. (V) braziliensis* (MHOM/CO/88/UA301-EGFP) in the dorsum. When exhibiting a skin ulcer greater than 25 mm^2^, they were randomly distributed into three experimental groups (*n* = 5 animals per group). Hamsters were treated topically with 100 µL per lesion per day of Arnica tincture during 28 days. Glucantime^®^ was administered by intralesional injection at 200 µg (100 µL) twice per week for four weeks. One group of hamsters remained untreated. After the end of treatment, animals were kept under observation for a period of 90 days. The area of the ulcer was measured every two weeks using an electronic caliper. The evaluation time points were: pretreatment day (TD0), end of treatment (TD28) and post treatment days (PTD) 30, 60 and 90, respectively. The effectiveness of each treatment was assessed comparing the lesion sizes prior to and after treatment for each individual. The outcome at the end of study was recorded as *cure* (epithelial healing and emergence of fur); *improvement* (reduction of lesion size by at least >20%); *relapse* (reactivation of lesion after initial improvement or cure); or *failure* (increase of lesion size). In addition, hamsters were weighed every two weeks from TD0 until PTD90.

It should be noted that the different lesion sizes in the various animals at the beginning of the treatment (Table 1) are due to the individual response of hamsters to infection. As human patients, each hamster develops a lesion different in size, typically 6–8 weeks after infection. It his hence not possible to obtain uniform lesion sizes within the groups. The results are therefore not averaged but presented individually for each animal.

The Ethics Committee for Animal Experimentation at the Universidad de Antioquia approved all experiments conducted with hamsters (Act No. 107 of 1 December 2016).

## 4. Conclusions

The overall results obtained with Arnica tincture in this study are at least qualitatively comparable with (or even somewhat better than) that of the standard drug. Despite a slower healing process obtained with Arnica tincture in this first study, the present results may hence be considered as a promising starting point for further studies. It should be noted that the amount of administered active constituents with known antileishmanial activity, i.e., STLs, was quite low in comparison with the antimonial (about 1.4 µg vs. 1600 µg total dose over 4 weeks) which emphasizes these compounds’ high potential as antileishmanials. Further studies will thus have to be conducted in order to optimize the dosage and treatment regimen (e.g., number of treatment repeats, occlusion of treated ulcers vs. treatment on open skin, application as liquid or gel or ointment and other variables). Furthermore, the potential of Arnica preparations will also be tested against further *Leishmania* species responsible for CL. It will also be of interest to investigate whether the total extract present in the tincture is superior in activity to isolated STL, i.e., helenalin and 11α,13-dihydrohelenalin esters. These compounds are known to possess antileishmanial activity [2,3,4] but it is quite likely that their proven anti-inflammatory activity [5,6,7] also contributes to the curative effects observed in the present CL model.

## Figures and Tables

**Table 1:** Lesion size in *L. braziliensis*-infected hamsters treated with Arnica tincture and meglumine antimoniate vs. untreated.

**Arnica Tincture**									
**Hamster Code**	**TD0**	**TD28**	**PTD15**	**PTD30**	**PTD45**	**PTD60**	**PTD75**	**PTD90**	**Outcome**
1AE-354-OD-♂	61.31	37.29	49.73	44.86	65.72	41.58	15.13	0.00	CURE
2AE-354-SM-♂	49.19	47.69	51.63	32.46	77.86	42.29	36.84	85.852 ^1^	IMPROVEMENT
3AE-356-OD-♂	40.30	51.87	96.46	69.52	95.83	43.73	14.81	0.00	CURE
4AE-357-SM-♀	36.58	0.00	2.81	16.96	11.36	4.72	2.75	0.00	CURE
5AE-358-SM-♀	65.52	0.00	18.59	42.68	50.00	40.84	22.68	12.33	RELAPSE
**Meglumine Antimoniate**									
**Hamster Code**	**TD0**	**TD28**	**PTD15**	**PTD30**	**PTD45**	**PTD60**	**PTD75**	**PTD90**	**Outcome**
1G-151-SM-♂	117.86	0.00	0.00	0.00	0.00	0.00	0.00	0.00	CURE
2G-153-OI-♂	143.72	0.00	0.00	0.00	12.57	12.57	0.00	12.57	RELAPSE
3G-155-OI-♂	70.14	102.07	0.00	14.52	7.07	12.57	39.37	10.18	RELAPSE
4G-157-OI-♀	160.38	97.12	0.00	0.00	0.00	0.00	0.00	0.00	CURE
5G-163-OD-♀	117.28	0.00	0.00	0.00	0.00	12.57	0.00	123.51	RELAPSE
**Untreated**									
**Hamster Code**	**TD0**	**TD28**	**PTD15**	**PTD30**	**PTD45**	**PTD60**	**PTD75**	**PTD90**	**Outcome**
1U-183-OI-♂	153.90	173.90	156.37	189.53	183.23	164.00	132.57	177.83	ACTIVE CL
2U-117-AO-♀	87.72	153.72	83.37	175.30	131.31	90.76	144.20	70.14	ACTIVE CL
3U-210-OI-♀	92.95	102.95	119.64	118.40	109.32	122.45	148.01	132.97	ACTIVE CL
4U-219-OD-♂	84.42	114.47	122.99	124.58	131.92	122.20	161.70	170.26	ACTIVE CL
5U-386-OD-♂	46.61	48.64	78.35	45.25	58.46	71.77	64.31	42.98	ACTIVE CL

^1^ The animal showed an improvement but was bitten by another hamster so that the lesion size that had previously been decreasing increased again on PTD90.

## Supplementary Materials

The supplementary materials are available online. Figures S1–S3: Photographs of all animals’ lesion at TD0, TD28 and PTD90 for both groups, treated with Arnica tincture and meglumine antimoniate as well as untreated control animals.
