# Supplementary material for: Arnica Tincture Cures Cutaneous Leishmaniasis in Golden Hamsters
Source: Molecules. 2018 Jan 12;23(1):150. doi: 10.3390/molecules23010150 (PMC6017635; doi:10.3390/molecules23010150)
Supplement: Supplementary file 1 [file molecules-23-00150-s001.pdf]

Supplementary Material

Arnica Tincture Cures Cutaneous Leishmaniasis in Golden Hamsters

Sara M. Robledo [1], Ivan D. Vélez [1] and Thomas J. Schmidt [2,*]

[1] PECET-School of Medicine, University of Antioquia, Calle 70 # 52-21, 0500100 Medellin , Colombia; sara_robledo@yahoo.com or sara.robledo@udea.edu.co (S.M.R.); id_velez@yahoo.com or ivan.velez@udea.edu.co (I.D.V.)

[2] Institute of Pharmaceutical Biology and Phytochemistry (IPBP), University of Münster, PharmaCampus-Corrensstrasse 48, D-48149 Münster, Germany

* Correspondence: thomschm@uni-muenster.de; Tel.: +49-251-83-33378

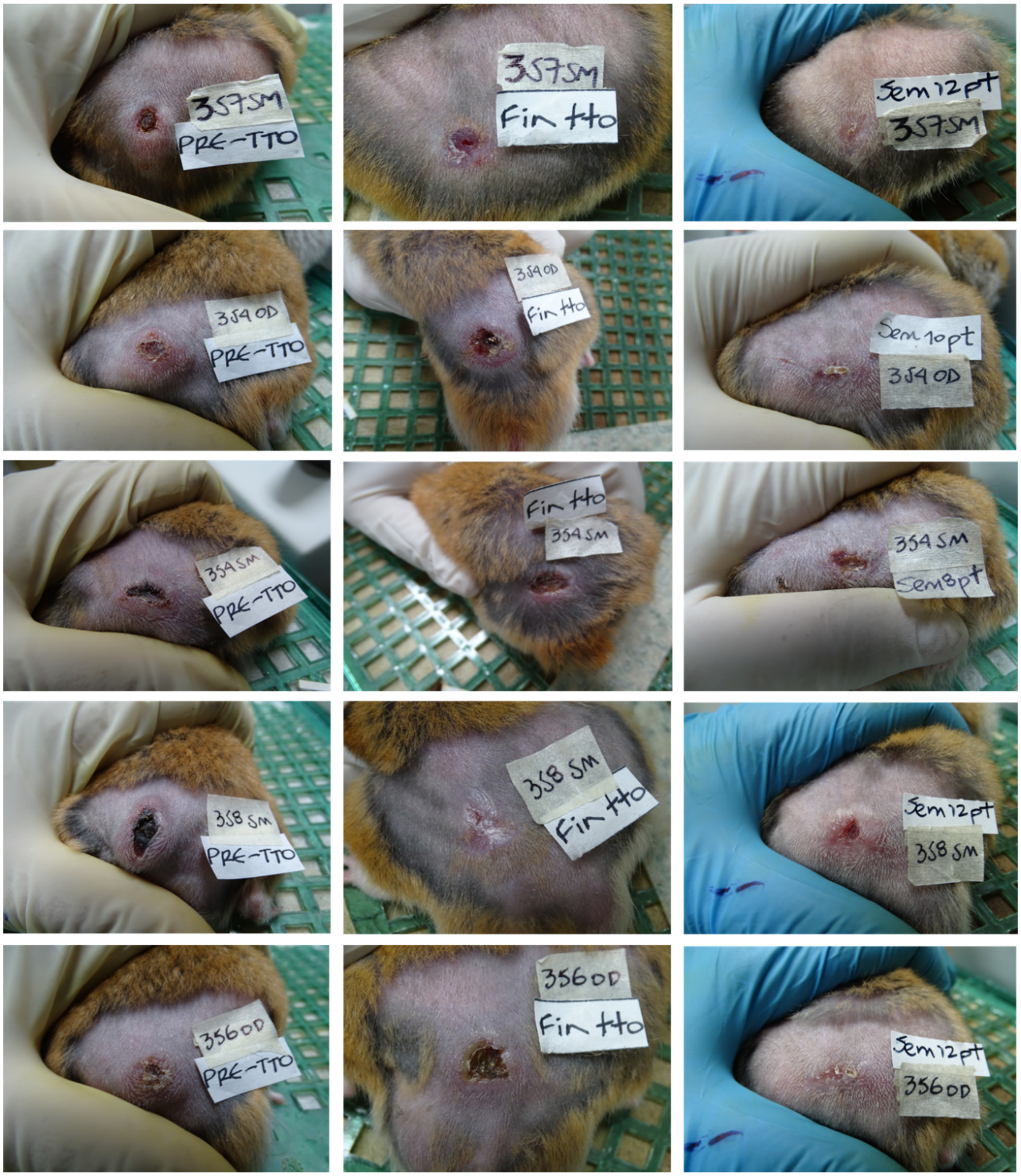

Figure S1. CL lesions in hamsters treated with Arnica tincture on day 0 (pre treatment; left), day 28 (end of treatment; middle) and on day 90 post treatment (PTD90; right). (The pictures shown in the top row are those shown in Figure 3 of the main document.).

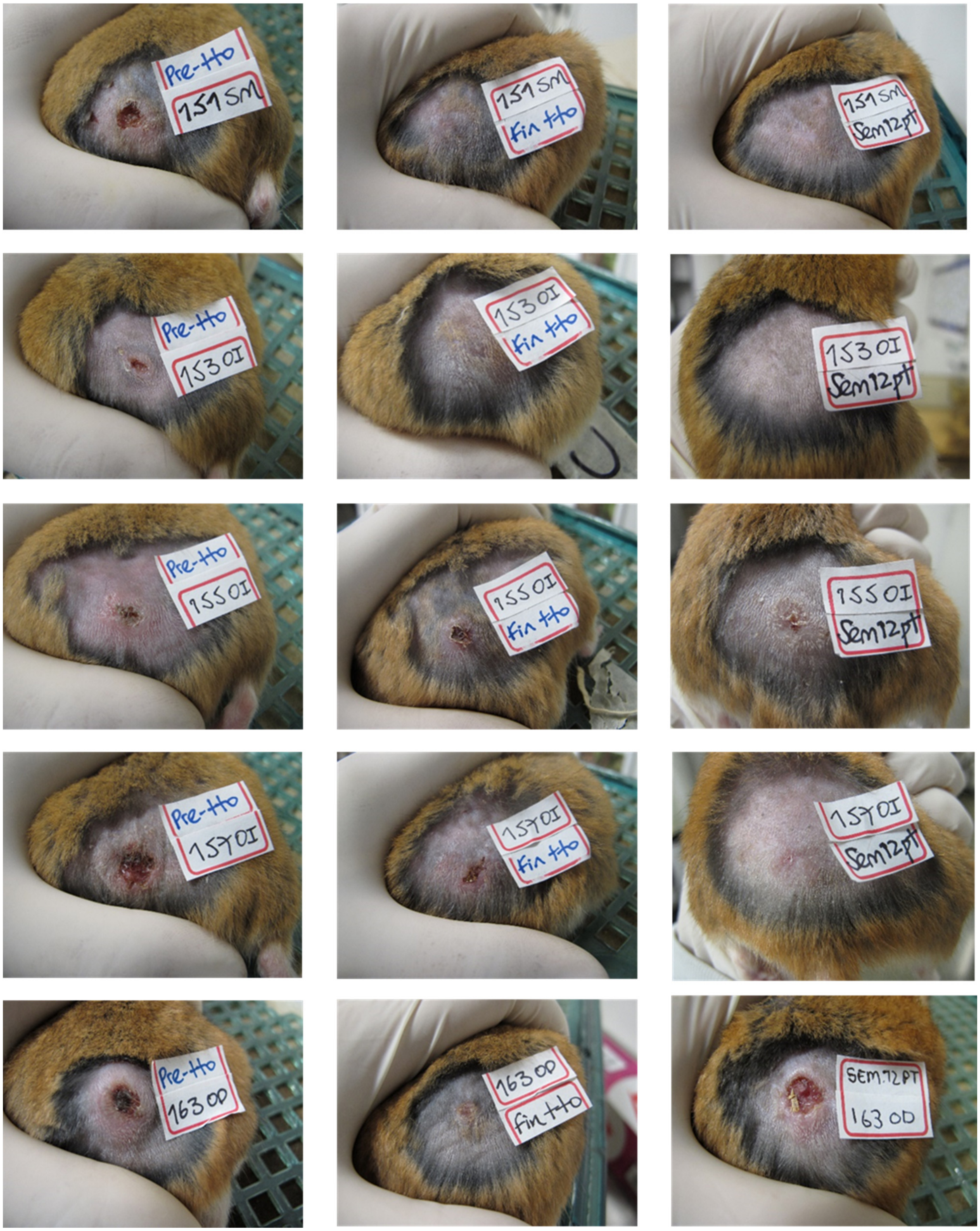

Figure S2. CL lesions in glucantime-treated hamsters (positive control) on day 0 (pre treatment; left), day 28 (end of treatment; middle) and on day 90 post treatment (PTD90; right).

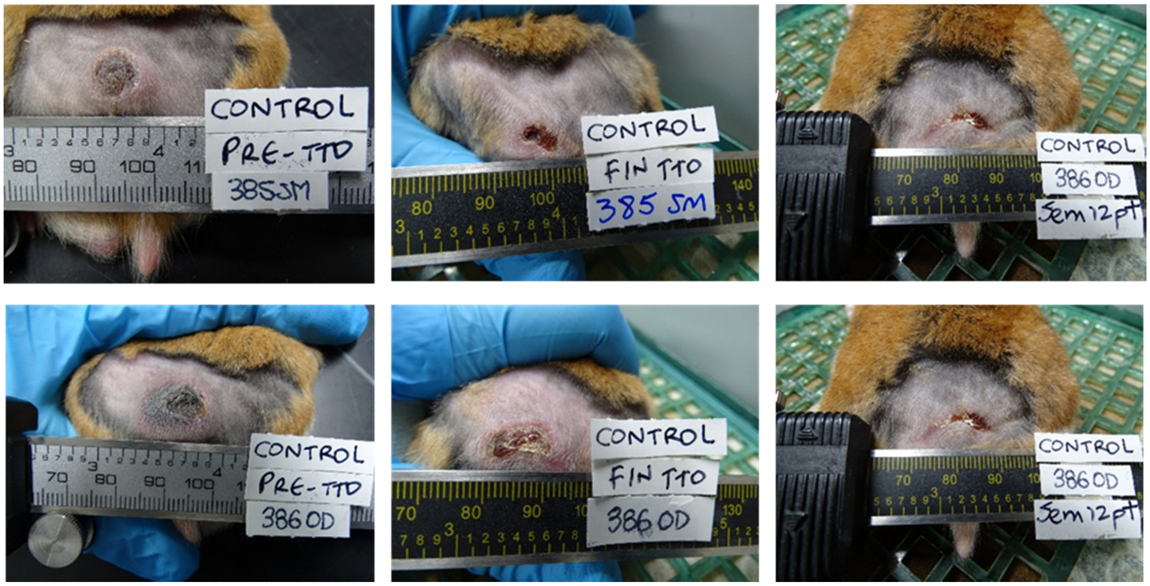

Figure S3. CL lesions in untreated hamsters (negative control) on day 0 (left), day 28 (middle) and on day 118 (right).
